# Incidence and risk factors of deep vein thrombosis after extracorporeal life support

**DOI:** 10.1111/aor.14271

**Published:** 2022-04-29

**Authors:** Olivier van Minnen, Walter M. van den Bergh, Joep M. Droogh, Lisette Koehorst, Wim K. Lagrand, S. Jorinde Raasveld, Annemieke Oude Lansink‐Hartgring, Aart Terpstra, Jasper M. Smit, Pieter R. Tuinman, Alexander P. J. Vlaar

**Affiliations:** ^1^ Department of Critical Care, University Medical Center Groningen University of Groningen Groningen The Netherlands; ^2^ Department of Radiology, Amsterdam University Medical Center (Location AMC) University of Amsterdam Amsterdam The Netherlands; ^3^ Department of Intensive Care Medicine, Amsterdam University Medical Center (Location AMC) University of Amsterdam Amsterdam The Netherlands; ^4^ Department of Intensive Care Medicine, Amsterdam University Medical Center (Location VUmc) Vrije Universiteit Amsterdam The Netherlands

**Keywords:** anticoagulant, deep vein thrombosis, extracorporeal life support

## Abstract

**Background:**

Deep vein thrombosis (DVT) after decannulation of extracorporeal life support (ECLS) is not uncommon. Moreover, the impact of anticoagulation and potential risk factors is unclear. Furthermore, it is unclear if cannula‐associated DVT is more common in ECLS patients compared to critically ill patients without ECLS.

**Methods:**

All adult patients who were successfully weaned from ECLS and were screened for DVT following decannulation were included in this observational cohort study. The incidence of post‐ECLS‐DVT was assessed and the cannula‐associated DVT rate was compared with that of patients without ECLS after central venous catheter (CVC) removal. The correlation between the level of anticoagulation, risk factors, and post‐ECLS‐DVT was determined.

**Results:**

We included 30 ECLS patients and 53 non‐ECLS patients. DVT was found in 15 patients (50%) of which 10 patients had a DVT in a cannulated vein. No correlation between the level of anticoagulation and DVT was found. V‐V ECLS mode was the only independent risk factor for post‐ECLS‐DVT (OR 5.5; 95%CI 1.16–26.41). We found no difference between the ECLS and non‐ECLS cohorts regarding cannula‐associated DVT rate (33% vs. 32%).

**Conclusion:**

Post‐ECLS‐DVT is a common finding that occurs in half of all patients supported with ECLS. The incidence of cannula‐associated DVT was equal to CVC‐associated DVT in critically ill patients without ECLS. V‐V ECLS was an independent risk factor for post‐ECLS‐DVT.

## Graphical abstract

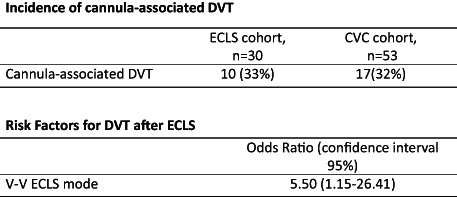

## 1 INTRODUCTION

In the past decades, the use of veno‐venous (V‐V) and veno‐arterial (V‐A) extracorporeal life support (ECLS) has increased.^1^ Although ECLS might be lifesaving in selected patient populations, mortality remains high. This is partly due to the irreversibility of the disease for which ECLS is initiated, but a significant part is a treatment‐related mortality.^2^ The most common complications associated with ECLS are bleeding and thrombosis which both have a significant impact on morbidity and mortality.^3^ Optimal anticoagulation therapy aiming to reduce the risk of thrombosis without inducing bleeding is a delicate balance. Currently, unfractionated heparin (UFH) is the most commonly used for therapeutic anticoagulation in ECLS‐patients, most commonly guided by activated partial thromboplastin time (aPTT) with a target of 1.5–2.5 times baseline (the actual target depends on local protocols).^4^ However, this aPTT target is adapted from other diseases or indications for therapeutic anticoagulation, and validation for ECLS is lacking. Moreover, the relationship between aPTT and thrombotic complications in ECLS patients is unclear, while higher aPTT is directly correlated with the incidence of hemorrhagic complications.^5^ Major bleeding is reported to be up to 45% in patients receiving ECLS.^5^ On the other hand, ECLS and its potential attendant prothrombotic inflammatory environment may increase the risk of thrombosis. Previous studies reported various incidences of deep vein thrombosis (DVT) after decannulation of ECLS, ranging from 18% to 85% depending on the type of screening method.^6^, ^7^, ^8^, ^9^, ^10^, ^11^

The clinical significance of DVT in ECLS patients is still unclear. In case ECLS‐related DVT is present, optimal treatment is speculative as follow‐up data are lacking in these patients. Also, a direct comparison between DVT patients in critically ill with and without ECLS has not been performed. Critically ill patients admitted to the ICU, in general, are at high risk of thrombosis, especially during sedation and mechanical ventilation.^12^ Thrombosis rates ranging from 5% to 31% in critically ill admitted to the ICU have been reported, although the presentation is mostly asymptomatic.^12^ The risk for DVT is increased when an indwelling central venous catheter (CVC) is present, and may also be size‐dependent which potentially puts ECLS patients at higher risk.^13^

Insight into the incidence and risk factors of ECLS‐associated large vessel thrombosis is highly needed. The objective of this study was to determine the incidence and risk factors of DVT after decannulation of ECLS (post‐ECLS‐DVT), to assess the relationship with the level of anticoagulation, and to compare the cannula‐associated DVT rate in patients with and without ECLS.

## 2 METHODS

We conducted an observational cohort study. Institutional approval was given for this study and the need for informed consent was waived. The study was performed at two sites of a closed format mixed medical‐surgical ICU in a Dutch tertiary referral hospital. The design and conduct of this study followed the STROBE checklist for cohort studies.^14^

All consecutive adult patients who received ECLS between January 2018 and March 2020 and were successfully weaned and decannulated were included. Routine venous duplex ultrasound for the evaluation of DVT was performed within 24 h following decannulation by a radiologist or trained laboratory technician. In ECLS patients we performed a routine venous duplex ultrasound of both jugular and femoral veins, limited to the neck and groin. In non‐ECLS patients, duplex ultrasound was limited to the vein in which the CVL was inserted. DVT was diagnosed by incompressibility of the vein, absence of flow, or presence of thrombus.

Additional data on demographics, anticoagulation, and ECLS characteristics were extracted from the electronic charting system (EPIC®, March 2019).

We compared baseline characteristics and DVT rate, in patients on ECLS with a prospective composed cohort of critically ill adult patients without ECLS who received a CVC for at least 48 h. This group was treated with a prophylactic dose of low molecular weight heparin. All patients were screened for DVT before and 24–48 h after CVC removal by color doppler and compression ultrasound examinations of the CVC entry vein by a trained researcher. Diagnosis of DVT was similar as described above for ECLS patients.

When DVT was detected, patients in both groups were treated with therapeutic dose anticoagulation for 3 months.

### 2.1 Institutional guidelines

Our standard adult V‐V ECLS circuit consisted of a Novalung® device and a dedicated X‐lung membrane (Xenios®). Heparin‐coated cannulas were inserted ultrasound‐guided, percutaneously, by a trained intensivist (Seldinger technique). The V‐A ECLS circuit consisted of the Cardiohelp® device (Maquet‐Getinge®) with dedicated ECLS cannulas and circuits (HLS Set Advanced 7.0®). Heparin‐coated cannulas were placed by a cardiothoracic surgeon using the open cutdown Seldinger technique. Anticoagulation therapy consisted of an initial bolus of 5000 U UFH. Afterward, anticoagulation targets consisted of an aPTT of 50–70 s for V‐A‐ and 45–60 s (reference value: 22–29 s) for V‐V ECLS. This target was maintained by 6‐hourly aPTT tests and stepwise adaption of continuous UFH infusion according to a nurse‐driven algorithm. In patients with severe bleeding complications, UFH was reduced or temporarily stopped.

### 2.2 Statistical analysis

All quantitative data were expressed as mean ± SD or median [interquartile range], and categorical variables were expressed as frequencies and percentages (*n*, %). ECLS patients with and without DVT were compared. DVT incidence and baseline characteristics of patients with ECLS were compared with critically ill patients without ECLS. Normally distributed continuous variables were compared using the Independent‐Samples *t*‐test. Non normally distributed variables were compared using Mann–Whitney *U* tests. Categorical variables were compared using Fisher's exact test, due to the expected small sample size in some analyses. The level of anticoagulation with UFH was assessed using aPTT values in three ways. First, the mean aPTT was calculated as the average of all aPTT measurements during ECLS. When the duration of ECLS exceeded 3 days, the days of cannulation and decannulation were excluded as UFH may not be optimized or already stopped at the time of aPTT measurements. Second, we measured the time‐weighted average (TWA) of aPTT with two different thresholds (<50 and <60 s) by calculating the percentage of days of ECLS with at least one measurement under the threshold, again with exception of the first and last day of ECLS in case duration of ECLS exceeded 3 days. Univariate logistic regression models were conducted to analyze the association of all baseline variables with post‐ECLS‐DVT yielding crude odds ratios (OR) with a corresponding 95% confidence interval (CI95%). Three multivariable logistic regression models were conducted to analyze the association of level anticoagulation and the occurrence of post‐ECLS‐DVT: (1) mean aPTT per 10 s increase; (2) TWA aPTT <50 s and (3) TWA aPTT <60 s. Each model was adjusted with all factors with a *p*‐value of <0.1 in the univariate analysis as well as age and sex yielding adjusted odds ratios with corresponding CI95%. To assess risk factors for post‐ECLS‐DVT we performed a stepwise logistic regression using the backward method, wherein the full model consisted of all clinical meaningful variables. All reported *p‐*values were two‐sided, a *p‐*value <0.05 was considered statistically significant. We had no missing data or loss to follow up. All statistical analyses were conducted using IBM SPSS® Statistics software, version 26 (IBM Corporation®, 2019).

## 3 RESULTS

**FIGURE 1:**
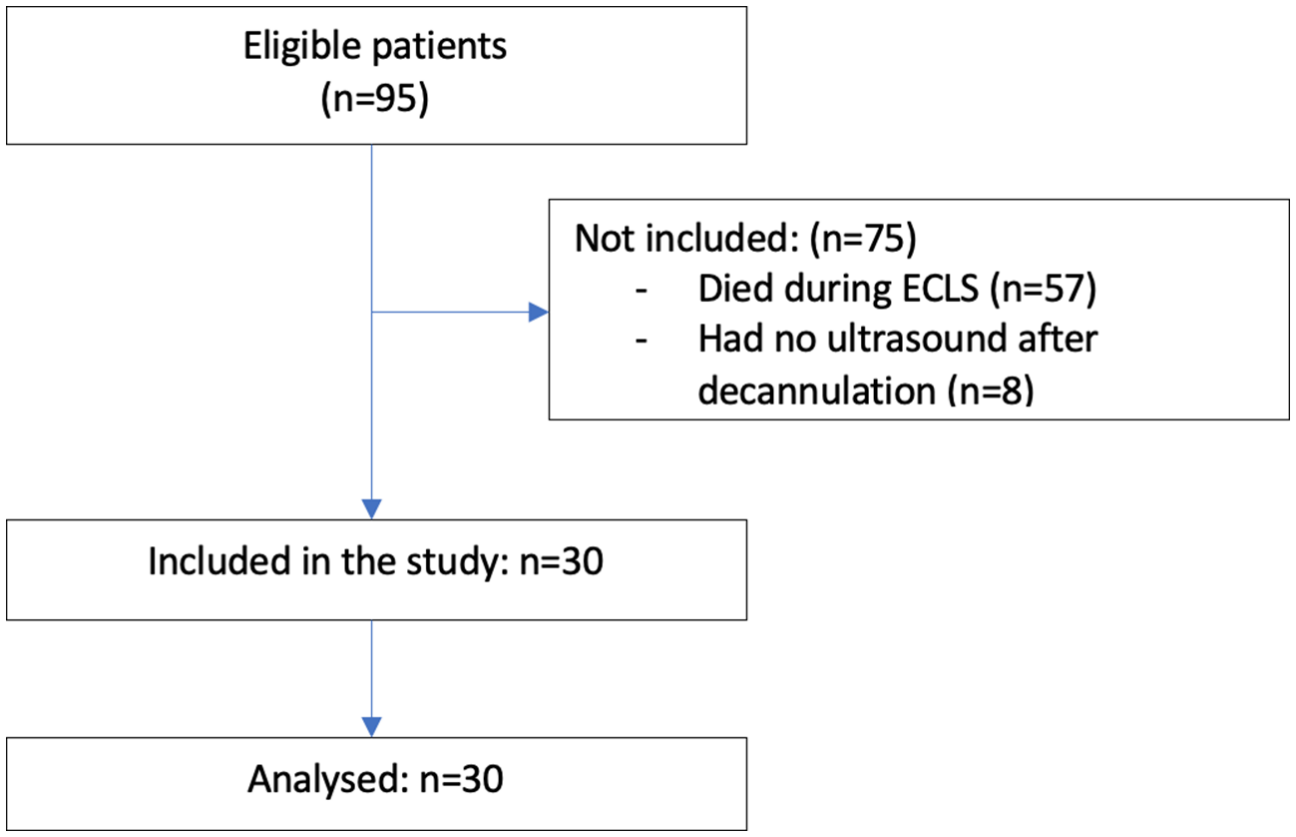
Flow diagram

During the study period, 95 patients were supported with ECLS. Thirty‐eight patients were successfully weaned from ECLS. Thirty patients (79%) were screened for post‐ECLS‐DVT by duplex sonography and included in the analyses. Figure 1 shows a flowchart depicting the inclusion of patients, as well as the reason for exclusion. The baseline characteristics of these 30 patients are presented in Table 1. The mean age was 50 years and 70% of the patients were male. Fourteen patients (47%) received VA ECLS. In comparison to patients on V‐V ECLS, patients on V‐A ECLS were older (56 vs. 44 years, *p* = 0.02), had a longer median duration of ECLS (6 vs. 5 days, *p* = 0.05), and higher mean aPTT value (59 vs. 47 s, *p* < 0.001). Fifteen patients (50%) had post‐ECLS‐DVT of which 10 patients had DVT in the cannulated vessel (cannula‐associated DVT). Patients with post‐ECLS‐DVT had a longer median duration of ECLS (6 vs. 4 days, *p* = 0.05) and were more often on V‐V ECLS (67% vs. 27%, *p* = 0.07) compared to patients without DVT. There were no significant differences in the level of anticoagulation between patients with and without post‐ECLS‐DVT (mean aPTT 51 vs. 54 s, *p =* 0.37), respectively.

**TABLE 1:** Baseline characteristics of study patients and outcome

	All *n* = 30	With DVT *n* = 15	Without DVT *n* = 15	*p* value
Male, *n* (%)	21 (70)	12 (80)	6 (40)	0.43
Age, years, mean (SD)	50 (±15)	46 (±15)	53 (±15)	0.10
Body mass index, kg/m^2^, mean (SD)	29 (±6)	30 (±7)	28 (±4)	0.72
Duration of ECLS, days mean [IQR]	5 [3–8]	6 [5–8]	4 [3–6]	0.05
Platelets, ×10^9^/L, mean (SD)	138 (±85)	155 (±93)	122 (±74)	0.48
aPTT, mean (SD)	52 (±10)	51 (±10)	54 (±11)	0.37
TWA APTT <50 s, median [IQR]	67 [50–100]	75 [50–100]	67 [50–100]	0.42
TWA APTT <60 s, median [IQR]	100 [100–100]	100 [100–100]	100 [88–100]	0.31
SOFA score, median [IQR]	12 [9–14]	12 [9–15]	12 [10–14]	0.67
Mode of ECLS, veno‐aterial, *n* (%)	14 (±47)	4 (±27)	10 (±67)	0.07
Mode of cannulation, peripheral, *n* (%)	29 (±97)	15 (100)	14 (±93)	1
Cannula drainage site, *n* (%)				0.52
LFV	7 (±23)	3 (20)	4 (±27)	
RFV	23 (±77)	12 (80)	11 (±73)	
Cannula return site, *n* (%)				0.13
Other	1 (±3)	0	1 (±7)	
LFA	3 (10)	0	3 (20)	
RFA	10 (±33)	4 (±27)	6 (40)	
RJV	15 (50)	10 (±67)	5 (±33)	
JVDL	1 (±3)	1 (±7)	0	
Respiratory diagnosis, *n* (%)				0.53
Pneumonia	10 (±33)	7 (±47)	3 (20)	
ARDS	2 (±7)	2 (±13)	0	
SA	4 (±13)	2 (±13)	2 (±13)	
Cardiac diagnosis, *n* (%)				0.26
MI	7 (±23)	3 (20)	4 (±27)	
Post‐cardiotomy	2 (±7)	1 (±7)	1 (±7)	
Myocarditis	1 (±3)	0	1 (±7)	
PE	4 (±13)	0	4 (±27)	
Accidental hypothermia with arrest	1 (±3)	1 (±7)	0	
Other	1 (±3)	1 (±7)	0	
Location DVT, *n* (%)				
DC		5 (±33)		
RC		3 (20)		
Other		5 (±33)		
DC and other		1 (±7)		
DC and RC		1 (±7)		

Abbreviations: aPTT, activated partial thromboplastin time; ARDS, acute respiratory distress syndrome; DC, drainage cannula; ECLS, extracorporeal life support; JVDL, jugular vein double lumen; LFA, left femoral artery; LFV, left femoral vein; MI, myocardial infarction; PE, pulmonary embolism; RC, returning cannula; RFA, right femoral artery; RFV, right femoral vein; RJV, right jugular vein; SA, status asthmaticus; TWA, time‐weighted average.

Factors associated with increased occurrence of post‐ECLS‐DVT in univariate analysis were V‐V ECLS mode (OR = 5.50, 95%CI 1.15–26.41) and longer duration of ECLS (OR = 1.35, 95%CI 0.83–1.86) (Table 2).

**TABLE 2:** Univariate odds ratio's for deep vein thrombosis after extracorporeal life support

	Crude OR (95% confidence interval)
Age, years	0.97 (0.92–1.02)
Male sex	2.67 (0.52–13.66)
Body mass index, kg/m^2^	1.06 (0.93–1.20)
Duration of ECLS, days	1.35 (0.83–1.86)
SOFA score	0.95 (0.76–1.19)
V‐V ECLS mode	5.50 (1.15–26.41)
Mean platelets, ×10^9^/L	1.01 (0.97–1.01)
Mean APTT per 10 s	0.76 (0.37–1.57)
TWA APTT <50 s	1.01 (0.99–1.04)
TWA APTT <60 s	1.05 (0.96–1.16)

Abbreviations: aPTT, activated partial thromboplastin time; ECLS, extracorporeal life support; TWA, time‐weighted average.

In the multivariable logistic regression analysis, we found no association between the level of anticoagulation and the occurrence of DVT (Table 3). Stepwise backward regression analysis showed V‐V ECLS mode to be the only independent risk factor for post‐ECLS‐DVT (OR = 5.50, 95%CI = 1.15–26.41).

**TABLE 3:** Multivariable logistic regression models

	Adjusted^a^ OR (confidence interval 95%)
Mean APTT per 10 s	1.25 (0.45–3.51)
TWA APTT <50 s	1.01 (0.98–1.04)
TWA APTT <60 s	1.05 (0.92–1.21)

Abbreviations: aPTT, activated partial thromboplastin time; ECLS, extracorporeal life support; TWA, time‐weighted average.

^a^ Adjusted for sex, age, ECLS‐mode, and duration of ECLS in days.

Baseline characteristics and DVT rate in the ECLS cohort were compared with those of 53 critically ill patients without ECLS, but with CVC indwelling for more than 48 h. The CVC cohort was older, had a longer catheter indwelling time, a higher mean platelet count, and a lower mean aPTT than the ECLS cohort. We found similar cannula‐associated DVT rates in the ECLS and CVC cohort (33% vs. 32%) (Table 4).

**TABLE 4:** Baseline characteristics of the cohort with a central venous catheter

	All (*n* = 53)
Male, *n* (%)	36 (67.9)
Age years, median [IQR]	62.0 [55.0–73.0]
BMI, kg/m^2^, mean (SD)	25.5 (4.6)
APACHE II score, mean (SD)	14.5 (6.4)
Insertion site CVC (%)	
IJV right	30 (57.7)
IJV left	8 (15.4)
SV right	3 (5.8)
SV left	1 (1.9)
FV right	4 (7.7)
FV left	6 (11.5)
Indwelling time, days, median [IQR]	7.0 [5.0–9.0]
Number of lumens (%)	
2	3 (5.9)
3	12 (23.5)
4	36 (70.6)
Platelets, ×10^9^/L, median [IQR]	221.0 [143.8–300.2]
aPTT, s, median [IQR]	37.5 [31.5–45.2]
INR, median [IQR]	1.2 [1.1–1.4]
Catheter‐related thrombosis (%)	
No	36 (67.9)
Yes	17 (32.1)

Abbreviations: aPTT, activated partial thromboplastin time; BMI, body mass index; CVC, central venous catheter; FV, femoral vein; IJV, internal jugular vein; INR, international normalized ratio; IQR, interquartile range; SD, standard deviation; SV, subclavian vein.

## 4 DISCUSSION

The major finding of our study is that post‐ECLS‐DVT is a frequent complication, occurring in half of the patients successfully weaned from ECLS. The level of anticoagulation is not associated with the occurrence of DVT. Regression analysis identifies V‐V ECLS as a risk factor for DVT. Pure ECLS cannula‐associated DVT was not more frequent than CVC‐associated DVT in critically ill non‐ECLS patients.

Screening for post‐ECLS‐DVT is not routinely performed in most hospitals. If screening is performed, the most commonly used diagnostic method is ultrasonographic imaging as in our study. Ultrasonography is a convenient, non‐invasive technique without radiation exposure. However, ultrasonography may underestimate the incidence of DVT due to technical limitations of imaging for pelvic and vena cava locations. In retrospective analyses in V‐V ECLS patients vena, cava thrombosis incidence of 51.7% was found at autopsy,^7^ An even higher incidence of 71% was found in a study where thoraco‐abdominopelvic CT‐scan was performed in 75 consecutive patients after decannulation of V‐V ECLS.^10^ This is considerably higher than the DVT rate we found and most probably caused by the screening method.

We found no association between the level of anticoagulation and the occurrence of post‐ECLS‐DVT which is in line with most other studies.^6^, ^8^, ^15^, ^16^ Some other studies, however, found an association between the level of anticoagulation and the occurrence of DVT.^7^, ^9^ In one study, the association between the level of anticoagulation and the occurrence of DVT was very small (OR per day 1.02, 95%CI 1.00–1.03).^9^ In contrast, a shorter mean aPTT (per second) in the same study was found to have a protective effect on DVT,^9^ which suggests that a higher level of anticoagulation does not prevent DVT.

Most studies included just one ECLS modality (V‐A or V‐V), which makes it difficult to compare them with our results, although the literature is consistent with our findings that DVT occurred more often in V‐V ECLS. The higher incidence in V‐V ECLS can be explained by several factors. V‐V ECLS uses two venous cannulas, which are bigger than arterial cannulas, which increased size may increase the risk of cannula‐associated DVT. The exact cannula size used for ECLS was not documented. In our center, cannulas ranging from 19 to 29 French are used for venous cannulation, and cannulas ranging from 15 to 21 French for arterial cannulation. Also, patients on V‐V ECLS had lower target aPTT at our institution. We found, however, no association between aPTT and the occurrence of post‐ECLS‐DVT.

The risk of developing DVT is already increased in critically ill patients, especially in patients in need of mechanical ventilation and sedation.^12^ Incidence is often underestimated due to the mainly asymptomatic occurrence of DVT.^17^ Previously conducted studies found DVT rates ranging from 5% to 31%^12^, ^17^, ^18^, ^19^, ^20^ with one study indicating that 93% of all thrombosis were asymptomatic.^20^ The use of CVC was designated as a risk factor for DVT in two studies.^19^, ^20^ Risk factors were internal jugular vein cannulation and therapeutic anticoagulation at the time of CVC insertion. The reason for the latter association may have been the hypercoagulable state for which the anticoagulation therapy was indicated according to the authors.^21^ Other postulated risk factors for DVT after CVC removal are: older age, CVC insertion in the subclavian vein, left‐sided CVC insertion, longer duration of a catheter in situ, and a catheter‐to‐vein ratio >0.45.^13^

The cannulas used for ECLS are larger, yielding a higher catheter‐to‐vein ratio resulting in an expected increased DVT rate compared to patients without ECLS. However, we found a similar catheter‐associated DVT rate in both groups, contradicting the prothrombogenic role of the ECLS. A note of caution is that non‐ECLS patients had a slightly longer indwelling time of CVCs so the actual risk may be less than compared to ECLS patients. On the contrary, the risk may be underestimated because aPTT was significantly less in non‐ECLS patients as UFH was not routinely administrated in that group. We, however, did not find an association between aPTT and the occurrence of DVT.

It is known that the formation of a connective tissue sleeve can develop after the indwelling of a CVC. This sleeve consists of cellular and non‐cellular components.^22^ It is not possible to distinguish between a connective tissue sleeve or thrombus with the use of B‐mode ultrasound. Therefore, we could have overestimated the incidence of DVT. It is not known if asymptomatic connective tissue sleeves or DVT after intravenous cannulation need treatment.^23^

This study has several limitations. Some of these are known limitations in observational studies, such as selection bias, incomplete datasets despite the prospective design, and lack of strict protocols in various areas. We used ultrasonography to routinely screen for post‐ECLS‐DVT. This is the safest and least invasive method. A disadvantage of this method is that DVT can be missed due to various factors such as DVT location and different interpretations between assessors despite the ample experience of the laboratory technicians. We limited our DVT screening to the neck and groin so a possible thrombus in the inferior (or superior) vena cava could, therefore, have been missed. Moreover, using a CT scan could disclose a higher incidence of DVT compared to ultrasound. Another limitation is possible selection bias: not all patients have been screened for post‐ECLS‐DVT. Patients who died during ECLS have not been screened for DVT. So, the actual incidence may be underestimated, and deceased patients may even have a thrombotic cause of death. For example, a DVT incidence of 39% in the autopsy was found in patients who died on V‐A ECLS,^24^ which is higher than the DVT rate in our patient group weaned from V‐A ECLS. We excluded 8 of the 38 ECLS survivors because no ultrasound examination was performed for various reasons, and if this exclusion was not at random, it may have resulted in selection bias. The most important limitation of this study is the small sample size.

Contrary to most studies we included both patients on V‐V and V‐A ECLS in one analysis. V‐V ECLS and V‐A ECLS were given using different devices. Moreover, aPTT target levels were different in V‐V ECLS and V‐A ECLS patients. Although we did not find an association between level of anticoagulation and post‐ECLS‐DVT we did find a relation between ECLS mode and post‐ECLS‐DVT, which makes it difficult to determine whether V‐V ECLS mode or the specific device with dedicated cannulas is a risk factor for post‐ECLS‐DVT. In a retrospective analysis, however, no difference in oxygenator change or clot‐forming between the devices used in our study was found.^25^ To analyze the influence of anticoagulation on post‐ECLS‐DVT, we included aPTT in three different analyses. Because of this, we postulate that we analyzed the association between the level of anticoagulation and post‐ECLS‐DVT in the best possible manner. We compared our results with a cohort of patients without ECLS but with a CVC from another location of our medical center. This makes a direct comparison difficult, especially regarding the potential effect of measured and unmeasured confounders on the outcome. We did not compare the severity of illness, e.g., APACHE scores, as not all patients started with ECLS on the first day of ICU admittance. However, it may be assumed that in general patients in need of ECLS are more critically ill compared to non‐ECLS patients. In the non‐ECLS cohort, ultrasound screening was done in the cannulated vessel only, thereby possibly underestimating the total DVT rate in that group.

## 5 CONCLUSION

DVT following ECLS is frequent with half the patients showing DVT after decannulation. However, similar cannula‐associated DVT rates in ECLS patients compared to CVC‐associated DVT rates in a non‐ECLS cohort of critically ill patients were found. We identified V‐V ECLS as an independent risk factor for post‐ECLS‐DVT. The level of anticoagulation based on aPTT values was not associated with the occurrence of post‐ECLS‐DVT. Further research is needed to reveal the clinical relevance of post‐ECLS‐DVT and factors associated with cannula‐associated DVT.

## AUTHOR CONTRIBUTIONS

Concept and design: Olivier van Minnen, Walter M. van den Bergh, Alexander P. J. Vlaar. Data analysis and interpretation: Olivier van Minnen, Walter M. van den Bergh, Joep M. Droogh, Annemieke Oude Lansink‐Hartgring, Wim K. Lagrand, S. Jorinde Raasveld, Jasper M. Smit, Pieter R. Tuinman, Alexander P. J. Vlaar. Critical revision of the article: Walter M. van den Bergh, Joep M. Droogh, Annemieke Oude Lansink‐Hartgring, Wim K. Lagrand, S. Jorinde Raasveld, Pieter R. Tuinman, Alexander P. J. Vlaar. Data collection: Olivier van Minnen, Annemieke Oude Lansink‐Hartgring, Jasper M. Smit, Aart Terpstra, Lisette Koehorst. Approval of the article: Olivier van Minnen, Walter M. van den Bergh, Joep M. Droogh, Lisette Koehorst; Wim K. Lagrand, S. Jorinde Raasveld, Annemieke Oude Lansink‐Hartgring, Aart Terpstra, Jasper M. Smit, Pieter R. Tuinman, Alexander P. J. Vlaar.

## CONFLICT OF INTEREST

The authors declare that they have no competing interests.

## ETHICS APPROVAL AND CONSENT TO PARTICIPATE

This study was approved by the Medical Ethics Review Committee of the Academic Medical Center, Amsterdam, The Netherlands. The requirement to obtain informed consent from patients enrolled in this study was waived.

## Data Availability

The datasets used and analyzed during the current study are available from the corresponding author on reasonable request.
